# The Effect of a Mindfulness-Based Class on Mindfulness, Achievement Emotions, Emotion Regulation, and Empathy in Nursing Students

**DOI:** 10.3390/nursrep15110374

**Published:** 2025-10-23

**Authors:** Mikyoung Lee

**Affiliations:** Department of Nursing, College of Korean Medicine, Dongshin University, Naju 58245, Republic of Korea; mikylee@dsu.ac.kr; Tel.: +82-61-330-4095

**Keywords:** mindfulness-based class, achievement emotions, emotion regulation, empathy, nursing students

## Abstract

**Background/Objectives**: While the benefits of mindfulness are well-documented, limited research has examined its specific impact on achievement emotions, emotion regulation, and empathy among nursing students. This study aimed to investigate the effect of a mindfulness-based class on mindfulness, achievement emotions, emotion regulation, and empathy in nursing students. **Methods**: A one-group, pretest–posttest design was employed. The participants were 93 second-year nursing students enrolled in a 15-week mindfulness-based class at a university in South Korea during the second semester of 2024. Self-reported measures of mindfulness, achievement emotions, emotion regulation, and empathy were collected at the beginning and end of the semester. Paired *t*-tests were conducted to compare the differences in study variables before and after the course. **Results**: The mindfulness-based class was significantly associated with increases in nursing students’ mindfulness, positive achievement emotions, reappraisal, and empathy, and decreases in negative emotions. Suppression showed no significant change. **Conclusions**: The findings indicate that mindfulness training can enhance emotional well-being and empathy, contributing to nursing students’ personal and professional growth. This highlights the potential benefits of integrating mindfulness education into nursing curricula to strengthen students’ resilience and improve patient-centered care.

## 1. Introduction

Nursing students frequently encounter substantial academic demands, clinical stress, and emotional challenges that can significantly influence their well-being and professional growth [1,2]. Due to the rigorous nature of nursing education, students often struggle with heightened stress, difficulties in managing emotions, and reduced empathy, which are crucial factors for effective patient care [3,4]. Therefore, identifying effective strategies to enhance nursing students’ emotional and cognitive resilience is important to support both their academic success and future professional competence.

Mindfulness-based interventions have gained attention as a promising approach to addressing these difficulties [5]. Mindfulness is defined as a state of heightened awareness and nonjudgmental acceptance of the present moment [6]. Studies suggest that mindfulness-based programs help students cultivate self-awareness, alleviate stress, and improve emotional regulation, enabling them to cope with the demands of nursing education more effectively [7]. Specifically, research in the nursing field has shown that mindfulness-based interventions can contribute to improved psychological well-being, stress management, and emotional regulation among both nursing students [3,5,8] and nurses [9,10].

Mindfulness also influences achievement emotions, which refer to emotions directly related to academic activities and outcomes, and they are crucial for motivation and academic success [11]. Positive achievement emotions, such as enjoyment and pride, are linked to enhanced engagement and perseverance in learning, while negative emotions like boredom and anxiety can hinder academic progress [12]. Numerous studies have shown that mindfulness increases positive achievement emotions by promoting cognitive flexibility and reducing emotional reactivity, while mitigating negative emotions [13,14]. In nursing education, research suggests that mindfulness significantly affects both positive and negative emotions among nursing students [3,4,5,15]. Moreover, mindfulness-based interventions have been found to effectively lower negative emotions in nurses, while improving their emotional well-being and job satisfaction [16].

Meanwhile, the relationship between mindfulness and emotion regulation is widely acknowledged in the literature. Mindfulness enhances emotion regulation by enabling individuals to recognize and manage their emotional responses without feeling overwhelmed [17]. Emotion regulation, defined as the ability to effectively manage and respond to emotional experiences, can play a vital role in promoting psychological well-being and professional competence [18,19,20]. Gross [18] outlines two primary emotion regulation strategies: reappraisal and suppression. Reappraisal involves cognitively reframing emotional events to modify their impact, allowing individuals to regulate emotions before they fully develop. This strategy is effective in reducing negative emotions and enhancing positive ones. In contrast, suppression focuses on inhibiting emotional expressions after they arise but does not lessen the intensity of negative emotions. Instead, it requires significant cognitive effort, which may limit its effectiveness in managing emotions.

Considering the emotionally demanding nature of nursing education, the ability to regulate emotions effectively is essential for maintaining well-being and ensuring high-quality patient care. Research indicates that individuals who practice mindfulness are better able to reframe negative emotions, leading to greater resilience and adaptability [21]. This is especially relevant for nursing students, as effective emotion regulation contributes to their ability to navigate complex clinical situations and maintain professional composure while caring for patients [22].

In addition, mindfulness is increasingly recognized for its beneficial influence on empathy, which is a fundamental element of patient-centered care [23,24]. Empathy plays a crucial role in nursing, as it is directly linked to influencing patient satisfaction, therapeutic relationships, and the overall quality of care [23,25]. Research suggests that mindfulness training can develop empathy by improving emotional regulation, alleviating cognitive overload, and helping nurses remain fully present with their patients [25]. Through the practice of mindfulness, nurses can become more aware of their own emotions and reactions, facilitating more empathetic and attentive interactions with patients [23]. By encouraging present-moment awareness and promoting a nonjudgmental perspective, mindfulness allows healthcare providers to engage more deeply with patients, ultimately reinforcing therapeutic relationships [26]. Furthermore, incorporating mindfulness into nursing practice may help to prevent compassion fatigue and emotional exhaustion, which are common challenges in nursing education and clinical settings [27].

Given these findings, integrating mindfulness-based courses into nursing education may offer a valuable means of improving students’ well-being, academic performance, and professional readiness. Despite growing evidence of the benefits of mindfulness, limited studies have explored its specific impact on achievement emotions, emotion regulation, and empathy in nursing students. Furthermore, while existing studies have focused on short-term mindfulness-based interventions [5], there is a need to investigate the effects of an extended mindfulness-based class on nursing students’ cognitive and emotional development. Addressing this gap, the present study aimed to investigate whether a structured mindfulness-based class would improve mindfulness, achievement emotions, emotion regulation, and empathy—critical psychological and professional attributes in nursing.

## 2. Materials and Methods

### 2.1. Research Design

The present study employed a one-group, pretest–posttest design to evaluate the effectiveness of a 15-week mindfulness-based class on mindfulness, achievement emotions, emotion regulation, and empathy in nursing students.

### 2.2. Participants and Procedure

The study included 93 second-year nursing students enrolled in a mindfulness-based class at a university in J province, South Korea, during the second semester of 2024. The course was a mandatory, for-credit class integrated into the curriculum. Attendance was systematically monitored, with an average attendance rate exceeding 90%. Although students were encouraged to engage in daily home practice (approximately 5–10 min per day), participation in these activities was voluntary. Adherence to home practice was not formally monitored or recorded. The participants were from two separate classes, which were both taught by the same instructor. Among the participants, 85 were female (91.4%) and 8 were male (8.6%). The average age was 20.02 years (SD = 0.92), with an age range of 18 to 28 years. Data were collected through an online survey after obtaining written online consent. Data collection consisted of a pre-survey conducted in Week 1 (September 2024) at the beginning of the semester, 15 weekly class sessions, and a post-survey administered in Week 14 (December 2024) at the end of the semester. A total of 93 students participated in both the pre- and post-surveys, with no dropouts throughout the study.

### 2.3. Intervention

The mindfulness-based class was led by an experienced nursing professor with a strong background in mindfulness research and teaching, who has delivered numerous mindfulness-based lectures to nursing students and healthcare professionals. This professor also served as the researcher in the present study and was actively engaged in both academic and practical mindfulness applications. Since the class was taught by the researcher, strict ethical safeguards were implemented to reduce potential coercion (see *Ethical Considerations* for details). The class was designed to provide nursing students with an in-depth understanding of mindfulness, emphasizing the development of attention, awareness, and acceptance as essential skills for both personal and professional growth. The course incorporated evidence-based mindfulness practices tailored specifically to the needs of nursing students, ensuring relevance to their future roles in healthcare.

The class was conducted for two hours each week over a 15-week period, with each session carefully structured to build progressively on the skills and concepts introduced in the previous week. The sessions included guided mindfulness practices, discussions on theoretical foundations, and reflections on how mindfulness could improve both self-care and patient care. At the end of each class, students were asked to write in their reflection journals, responding to three key questions: What are the key takeaways from today’s class? What thoughts or feelings arose during the class? How would you apply what you learned today to your daily life? This reflective practice encouraged students to integrate mindfulness into their daily routines and deepen their understanding of the material. The detailed contents and structure of the 15-week mindfulness-based class are presented in Table 1, providing a comprehensive overview of the curriculum and session objectives.

### 2.4. Measures

The level of mindfulness among nursing students was assessed using the Cognitive and Affective Mindfulness Scale—Revised (CAMS-R). This measure was originally developed by Feldman et al. [28] and later adapted and validated for Korean populations by Cho [29]. The CAMS-R consists of three key dimensions: attention (4 items), awareness (4 items), and acceptance (2 items). Higher total scores indicate greater mindfulness. Participants provided their answers using a four-point Likert scale, where 1 represented “not at all” and 4 indicated “almost always”. The internal consistency of the original CAMS-R [28] was Cronbach’s α = 0.77, while the Korean version demonstrated a reliability coefficient of α = 0.70 [29]. In the present study, the overall reliability was α = 0.71, with sub-scale reliabilities of 0.73 for attention, 0.65 for awareness, and 0.62 for acceptance.

This study measured nursing students’ achievement emotions using the Achievement Emotions Questionnaire (AEQ), initially developed by Pekrun et al. [30]. For this research, the Korean-adapted and validated version by Lee [31] was used. The AEQ assesses three positive emotions (enjoyment, hope, and pride) and five negative emotions (boredom, anger, anxiety, hopelessness, and shame), comprising 12 items for positive emotions and 20 items for negative emotions. Participants responded on a five-point Likert scale, with one representing “strongly disagree” and five representing “strongly agree”. The internal consistency of the scale was strong, with Cronbach’s α = 0.84 for positive emotions and 0.86 for negative emotions, confirming its reliability.

This study assessed participants’ use of emotion regulation strategies through the Korean-adapted version of the Emotion Regulation Questionnaire (ERQ), which was originally developed by Gross and John [20] and validated in Korean by Shon [32]. The ERQ evaluates two key strategies: reappraisal (6 items) and suppression (4 items). Participants rated their responses on a five-point Likert scale, extending from one (strongly disagree) to five (strongly agree), with higher scores reflecting a stronger tendency to employ emotion regulation strategies in each domain. In the original version, the Cronbach’s α values for reappraisal and suppression were 0.82 and 0.76 [20], while the Korean adaptation reported 0.85 and 0.73 [32], respectively. In this study, the internal reliability was 0.81 for reappraisal and 0.70 for suppression, confirming acceptable consistency.

To assess the empathy level among nursing students, this study utilized the Korean-adapted version of the Interpersonal Reactivity Index (IRI), originally developed by Davis [33] and later validated by Kang et al. [34]. The IRI employs a multidimensional framework to evaluate both cognitive and emotional aspects of empathy. The cognitive empathy component includes perspective-taking and fantasy, while the emotional empathy component encompasses empathetic concern and personal distress. The instrument consists of 28 items, with each sub-scale containing 7 items. Participants provided responses using a five-point Likert scale, where one indicated “strongly disagree” and five indicated “strongly agree”; higher scores reflected greater empathy within each domain. The original IRI demonstrated sub-scale reliability coefficients, for Cronbach’s α, ranging from 0.68 to 0.79 [33], whereas the Korean version reported an overall Cronbach’s α of 0.80, with sub-scale values between 0.61 and 0.81 [34]. In this study, the total scale demonstrated a Cronbach’s α of 0.82, with 0.81 for cognitive empathy and 0.67 for emotional empathy, indicating acceptable internal consistency.

Although some sub-scales demonstrated modest internal consistency (e.g., CAMS-R acceptance, α = 0.62; IRI emotional empathy, α = 0.67), the satisfactory total-scale reliabilities (α = 0.71 and α = 0.82, respectively) support the adequacy of these instruments for the present study.

### 2.5. Data Analysis

The data were analyzed using SPSS version 28.0 (IBM Corp., Armonk, NY, USA). Initially, the participants’ demographic information was examined using means and standard deviations. To assess the relationships between mindfulness, achievement emotions, emotion regulation, and empathy after the mindfulness-based class, Pearson’s correlation coefficient was calculated. Additionally, paired *t*-tests were conducted to compare the differences in mindfulness, achievement emotions, emotion regulation, and empathy before and after the mindfulness-based class.

### 2.6. Ethical Considerations

The study was conducted in accordance with the principles of the Declaration of Helsinki. According to Article 2 of the Enforcement Rule of the Bioethics and Safety Act (Ministerial Ordinance No. 1048) and Article 2 of the Higher Education Act in Korea, research conducted as part of regular educational practices in designated institutions announced by the Minister of Health and Welfare does not require prior ethical approval. As this study involved the evaluation of an educational program conducted as part of routine university instruction, exemption under these statutes was automatic. Institutional permission for this study was granted by the head of the Department of Nursing. To ensure the full protection of participants’ rights and to minimize any sense of coercion or perceived pressure to participate, strict ethical safeguards were implemented. The study’s objectives, significance, and detailed procedures were thoroughly explained before data collection, allowing participants to make an informed decision regarding their involvement. Written informed consent was then obtained, emphasizing that participation was entirely voluntary. Participants were reassured that choosing not to participate would not result in any form of penalty, disadvantage, or negative consequence. To further safeguard autonomy, they were told that alternative non-research reflective activities would be available for those who preferred not to join the study. In addition, procedures were implemented to clearly separate instruction from research activities to minimize any grade-related pressure; participation remained voluntary and unrelated to course evaluation. Participants were further informed that all responses would remain strictly anonymous and confidential, and that the collected data would be used solely for research purposes. These measures were designed to uphold ethical standards and create a safe and transparent environment for participation.

## 3. Results

### 3.1. Correlations Between Study Variables

Table 2 presents correlation analysis results between the study variables. Correlations were examined cross-sectionally at post-intervention, consistent with the study’s aim of exploring relationships between the constructs following participation in the mindfulness-based class. Mindfulness showed a strong positive association with positive achievement emotions (r = 0.606, *p* < 0.001), whereas it was negatively related to negative achievement emotions (r = −0.501, *p* < 0.001). Moreover, mindfulness was positively linked to reappraisal, which is an emotion regulation strategy (r = 0.292, *p* < 0.01), while demonstrating a negative correlation with suppression (r = −0.213, *p* < 0.05). Empathy demonstrated a significant positive correlation with both mindfulness (r = 0.306, *p* < 0.01) and positive achievement emotions (r = 0.482, *p* < 0.001), but a negative association with negative achievement emotions (r = −0.220, *p* < 0.05). Additionally, reappraisal was positively associated with both positive achievement emotions (r = 0.542, *p* < 0.001) and empathy (r = 0.516, *p* < 0.001), while exhibiting a negative correlation with negative achievement emotions (r = −0.211, *p* < 0.05). Meanwhile, suppression was negatively related to both positive achievement emotions (r = −0.248, *p* < 0.05) and mindfulness (r = −0.213, *p* < 0.05), but had a positive relationship with negative achievement emotions (r = 0.372, *p* < 0.001).

### 3.2. Differences in Mindfulness, Achievement Emotions, Emotion Regulation, and Empathy

A paired *t*-test was performed to assess differences in mindfulness, achievement emotions, emotion regulation, and empathy before and after participation in the mindfulness-based class. The findings are presented in Table 3. Because the primary outcomes were prespecified (mindfulness total, positive/negative achievement emotions, reappraisal, suppression, and empathy total), unadjusted *p*-values for these outcomes are reported. Sub-scale analyses were exploratory and are presented with Cohen’s d effect sizes and 95% confidence intervals to facilitate interpretation.

The analysis revealed a significant increase in students’ mindfulness levels following the mindfulness-based class, with mean scores rising from 2.49 to 2.86 (*t* = −7.30, *p* < 0.001). Examining the subcomponents of mindfulness, attention improved significantly from 2.56 to 2.90 (*t* = −5.36, *p* < 0.001), awareness increased from 2.40 to 2.84 (*t* = −6.67, *p* < 0.001), and acceptance showed a notable rise from 2.49 to 2.83 (*t* = −3.98, *p* < 0.001).

Regarding achievement emotions, positive emotions significantly increased from 3.19 to 3.68 (*t* = −9.65, *p* < 0.001), whereas negative emotions significantly decreased from 2.70 to 2.44 (*t* = 5.05, *p* < 0.001). For emotion regulation, reappraisal significantly improved from 3.61 to 3.84 (*t* = −2.89, *p* = 0.005), while suppression showed no significant change (*t* = −0.04, *p* = 0.968).

Lastly, empathy significantly increased from 3.47 to 3.61 (*t* = −4.38, *p* < 0.001). Within its subcategories, cognitive empathy improved from 3.52 to 3.72 (*t* = −4.49, *p* < 0.001), and emotional empathy increased from 3.43 to 3.51 (*t* = −2.43, *p* = 0.017).

## 4. Discussion

This study examined whether a structured mindfulness-based class could improve mindfulness, achievement emotions, emotion regulation, and empathy among nursing students. The results showed that participation in mindfulness-based class was significantly associated with increases in nursing students’ mindfulness, positive achievement emotions, reappraisal, and empathy, as well as decreases in negative emotions, while suppression remained unchanged. These findings suggest that mindfulness training can enhance emotional well-being and empathy, contributing to nursing students’ personal and professional growth.

The present findings indicate that the mindfulness-based class significantly improved nursing students’ mindfulness levels. This aligns with previous research, suggesting that structured mindfulness interventions effectively cultivate mindfulness skills in students [6,35]. Several nursing studies have also demonstrated the positive effects of mindfulness-based interventions on mindfulness levels. For example, van der Riet et al. [2] found that a structured mindfulness program significantly enhanced nursing students’ mindfulness while reducing the negative emotion of anxiety. Similarly, Song and Lindquist [36] reported that nursing students who participated in mindfulness training exhibited improved attention and greater overall mindfulness compared to those who did not. These findings suggest that mindfulness education can be an effective strategy for enhancing self-awareness and emotional resilience among nursing students.

Furthermore, the structured nature of the mindfulness-based class may have played a crucial role in fostering mindfulness by providing students with essential tools to enhance awareness, attention, and acceptance—key components of mindfulness [37]. Regular engagement in mindfulness practices, such as focused breathing, body scan exercises, and mindful observation, likely facilitated students’ ability to remain present and respond to stressors more adaptively [37]. This is consistent with previous studies indicating that mindfulness training helps nursing students develop greater emotional stability and attentiveness, which are critical for managing the demands of clinical practice [9,10]. By incorporating mindfulness techniques into their daily routines, students may experience long-term benefits, including reduced emotional distress and improved overall well-being, highlighting the value of integrating mindfulness education into nursing curricula.

Regarding achievement emotions, participation in the mindfulness-based class was significantly associated with increased positive achievement emotions and decreased negative emotions among nursing students. This result is consistent with previous research showing that mindfulness training fosters more positive emotional experiences in academic settings [2,38]. In these studies, engaging in mindfulness techniques, such as deep breathing and nonjudgmental awareness, may have enabled students to handle academic difficulties more effectively, fostering greater confidence, enjoyment, and interest. Similarly, Song and Lindquist [36] found that mindfulness-based interventions in nursing education enhanced students’ ability to maintain a positive mindset, reducing depression and anxiety associated with challenging coursework.

The decrease in negative achievement emotions observed in this study may be attributed to mindfulness’s role in reducing rumination and self-criticism [39]. Previous research suggests that mindfulness helps nursing students remain present and approach academic challenges with greater composure, thereby alleviating stress-related emotions such as anxiety, frustration, and hopelessness [7,40]. By cultivating mindfulness through self-awareness and acceptance, students may have developed a healthier perspective on academic performance, allowing them to navigate setbacks with resilience [4,41,42]. Given the strong connection between emotional well-being and academic success [4,12], integrating mindfulness training into nursing curricula can contribute to a more supportive and engaging learning environment, improving students’ achievement emotions, motivation, and abilities to cope with the challenges of nursing education.

With respect to emotion regulation, participation in the mindfulness-based class was significantly related to increased use of reappraisal whereas suppression remained unchanged. Reappraisal, which is a strategy that involves reframing situations to alter their emotional impact, is closely associated with mindfulness, as both emphasize awareness and acceptance of experiences without immediate reaction [43,44]. Prior studies suggest that mindfulness training enhances individuals’ ability to engage in reappraisal by fostering a nonjudgmental awareness of thoughts and emotions, thereby enabling more adaptive emotional responses [22,43]. In nursing education, where students frequently encounter stressful situations, strengthening reappraisal skills through mindfulness may help them better regulate emotions, manage patient interactions, and maintain psychological well-being [38].

In contrast, the absence of significant change in suppression suggests that mindfulness practice alone may not be sufficient to alter habitual tendencies to inhibit emotional expression. Suppression, which involves deliberately reducing outward emotional displays, has been linked to increased psychological distress and decreased interpersonal communication effectiveness [18,19,20]. Since mindfulness encourages open awareness and acceptance of emotions rather than their avoidance or suppression [6], it is possible that students maintained their existing suppression tendencies despite improvement in reappraisal. Similar findings have been observed in previous studies, where mindfulness training primarily influenced reappraisal rather than response-focused regulation techniques such as suppression [45]. These results suggest that integrating additional emotion regulation strategies alongside mindfulness training in nursing curricula may be essential for fostering a more balanced approach to emotional expression and management. Specifically, future curricular adaptations could incorporate assertive communication, emotion-labeling, and experiential exercises that provide students with safe opportunities to practice expressing emotions.

Finally, participation in the mindfulness-based class was significantly associated with higher levels of empathy. This result is consistent with previous studies highlighting the relationship between mindfulness and empathy among healthcare professionals [26,27]. The significant increase in empathy following the mindfulness intervention suggests that mindfulness training may enhance nursing students’ ability to understand and respond to others’ emotions with greater sensitivity and compassion [27]. Mindfulness fosters present-moment awareness and nonjudgmental acceptance, which can help individuals become more attuned to both their own emotions and those of others, thereby strengthening empathetic engagement [35]. Previous research has demonstrated that mindfulness-based interventions enhance emotional regulation and reduce negative emotions, which in turn may facilitate deeper empathetic connections [7,25]. In the context of nursing education, where emotional resilience and interpersonal sensitivity are crucial, these findings align with studies suggesting that mindfulness training improves nursing students’ ability to provide compassionate, patient-centered care [2]. By fostering self-awareness and emotional balance, mindfulness may help students better manage the emotional demands of clinical practice, ultimately enhancing their ability to empathize with patients.

The increase in empathy also suggests that mindfulness practices may have facilitated students’ awareness of their own emotional states, enabling them to engage more deeply with patients’ emotions while maintaining professional boundaries [26,46]. Furthermore, mindfulness training can reduce burnout and emotional exhaustion, which are common barriers to empathetic engagement in nursing [27]. As empathy is strongly linked to improved patient outcomes, enhanced communication, and greater patient satisfaction [23], integrating mindfulness-based education into nursing curricula may provide long-term benefits for both students and the patients they serve.

Although this study provides valuable insights into the effects of a mindfulness-based class on nursing students, some limitations should be acknowledged. First, while the one-group, pretest–posttest design allows for within-group comparisons, the lack of a control group prevents ruling out alternative explanations for the observed changes. Future research should adopt a randomized controlled trial design to compare mindfulness-based interventions with other educational strategies or non-intervention groups, thereby strengthening the validity of the findings. Although improvements in emotion regulation and empathy may contribute to patient-centered care, the present study did not directly assess clinical or behavioral outcomes; thus, these potential links should be considered hypotheses to be tested in future controlled studies with clinical endpoints.

Second, several potential threats to internal validity should be considered. Maturation and testing effects cannot be ruled out, as improvements over time may partly reflect natural developmental processes or repeated exposure to the assessment instruments rather than the intervention itself. Demand characteristics may also have influenced participants’ responses, particularly given that the course was taught by a single instructor, which may have introduced expectancy effects. Moreover, although the absence of attrition strengthens the completeness of the data, it may also suggest subtle pressures to remain in the study. While modeling change scores controlling for baseline and class section could provide additional insights, the limited number of sections and the scope of this educational evaluation constrained the ability to conduct such analyses. Future studies with larger samples and multiple instructors may be better positioned to address these issues. Another limitation concerns the reliance on self-reported measures, which are susceptible to social desirability and subjective bias. Future studies could incorporate physiological indicators such as heart rate variability or structured behavioral assessments to provide more objective evaluations of mindfulness and emotional outcomes.

Additionally, individual variability in engagement with the mindfulness practices warrants consideration. Although all students participated in the structured class, their practice outside the sessions was not systematically monitored. The course was mandatory, and attendance rates exceeded 90%; however, engagement in daily home practice (approximately 5–10 min per day) was voluntary, and adherence to these practices was not formally assessed. Future research should assess adherence to mindfulness exercises and explore whether higher engagement levels lead to greater improvements in mindfulness, achievement emotions, emotion regulation, and empathy. Furthermore, while this study examined immediate post-intervention effects, the long-term sustainability of these benefits remains unclear. Longitudinal studies tracking students’ mindfulness and emotional competencies after graduation would help to determine whether mindfulness training has lasting impacts on professional practice and patient care.

## 5. Conclusions

This study examined the effects of a mindfulness-based class on nursing students’ mindfulness, achievement emotions, emotion regulation, and empathy. The findings demonstrated that participation in the mindfulness-based class was significantly associated with higher levels of mindfulness and empathy, along with increased positive achievement emotions and decreased negative emotions. Additionally, reappraisal was positively related to participation in the intervention, suggesting that mindfulness training may support adaptive emotion regulation strategies in nursing students. These results highlight the potential benefits of integrating mindfulness education into nursing curricula to foster emotional resilience and interpersonal sensitivity, both of which are critical for patient-centered care.

By strengthening students’ ability to manage emotions and connect empathetically with patients, mindfulness training can play a vital role in preparing future nurses for the emotional and psychological demands of clinical practice. Incorporating mindfulness-based education into nursing programs may not only enhance students’ well-being but also contribute to the development of more compassionate and effective healthcare professionals. As mindfulness continues to gain recognition in healthcare education, further efforts to refine and expand its application will help to maximize its benefits for both students and the patients they serve.

## Figures and Tables

**Table 1:** The contents of the 15-week mindfulness-based class.

Week	Topic	Contents
1	Orientation	Introduction to the mindfulness-based class: objectives, structure, assignments, evaluation criteria, and guidelines. Establishing class norms (ground rules).
2	Fundamentals of mindfulness	Core principles of mindfulness, its relationship with meditation, and the psychological and physiological benefits of mindfulness practice.
3	Being in the present moment	Cultivating present-moment awareness, sensory awareness, and mindfulness breathing techniques.
4	Mindfulness meditation 1	Introduction to mindfulness meditation: focused attention, the effects of meditation, and breath meditation practice.
5	Mindfulness meditation 2	Body scan, loving-kindness meditation, and walking meditation. Practice of meditation techniques.
6	Mindfulness and gratitude	The impact of gratitude on the brain and well-being. Mindfulness via gratitude. Gratitude journaling and reflective writing exercises.
7	Cultivating positivity	Introduction to positive psychology, characteristics of happy individuals, and character strengths assessment.
8	Mid-term assignment	Writing an essay on “My personal experiences applying mindfulness in daily life”.
9	Self-awareness and self-understanding	Recognizing personal weaknesses and shortcomings, recognizing emotional patterns, exploring core values in life, and engaging in mindful dialog with myself.
10	Understanding emotions	Utilizing mood meters, expressing own emotions, and managing emotions via mindfulness.
11	Emotion regulation	Strategies for stress management, emotion regulation strategies, and practicing meta-moment exercises.
12	Mindfulness and empathy	Developing empathetic communication, practicing empathy meditation, and active listening skills.
13	Mental muscles training 1	Inner-communication training for enhancing mental muscles: the origins of mental muscles, neuroplasticity, communication skills, and breath meditation.
14	Mental muscles training 2	Inner-communication training for enhancing mental muscles: gratitude (self-affirmation, affirmation of others), gratitude meditation, and forgiveness meditation. Reflection on the mindfulness-based class using photovoice methodology.
15	Final assignment	Class review and submission of the reflection note.

**Table 2:** Correlations between the study variables after the mindfulness-based class.

Variables	1	2.1	2.2	3.1	3.2	4
1. Mindfulness	1					
2. Achievement emotions						
2.1. Positive emotions	0.606 ***	1				
2.2. Negative emotions	−0.501 ***	−0.641 ***	1			
3. Emotion regulation						
3.1. Reappraisal	0.292 **	0.542 ***	−0.211 *	1		
3.2. Suppression	−0.213 *	−0.248 *	0.372 ***	0.132	1	
4. Empathy	0.306 **	0.482 ***	−0.220 *	0.516 ***	0.013	1

* *p* < 0.05, ** *p* < 0.01, *** *p* < 0.001.

**Table 3:** Differences in mindfulness, achievement emotions, emotion regulation, and empathy.

Variables		Pre	Post	*t*	*p*	Cohen’s *d* [95% CI]
		M ± SD				
Mindfulness		2.49 ± 0.38	2.86 ± 0.43	−7.30	<0.001	0.50 [−0.99, −0.52]
	Attention	2.56 ± 0.56	2.90 ± 0.58	−5.36	<0.001	0.61 [−0.77, −0.34]
	Awareness	2.40 ± 0.52	2.84 ± 0.50	−6.67	<0.001	0.63 [−0.92, −0.46]
	Acceptance	2.49 ± 0.64	2.83 ± 0.73	−3.98	<0.001	0.82 [−0.62, −0.20]
Achievement emotions	Positive emotions	3.19 ± 0.50	3.68 ± 0.49	−9.65	<0.001	0.49 [−1.25, −0.75]
	Negative emotions	2.70 ± 0.53	2.44 ± 0.62	5.05	<0.001	0.49 [0.31, 0.74]
Emotion regulation	Reappraisal	3.61 ± 0.66	3.84 ± 0.66	−2.89	0.005	0.74 [−0.51, −0.09]
	Suppression	3.03 ± 0.77	3.03 ± 0.88	−0.04	0.968	0.64 [−0.21, 0.20]
Empathy		3.47 ± 0.41	3.61 ± 0.41	−4.38	<0.001	0.31 [−0.67, −0.24]
	Cognitive empathy	3.52 ± 0.55	3.72 ± 0.51	−4.49	<0.001	0.31 [−0.67, −0.24]
	Emotional empathy	3.43 ± 0.36	3.51 ± 0.40	−2.43	0.017	0.32 [−0.46, −0.05]

## Data Availability

The data presented in this study are available from the author upon request due to privacy considerations.

## Public Involvement Statement

No public involvement in any aspect of this research.

## Guidelines and Standards Statement

This manuscript was drafted against the TREND (Transparent Reporting of Evaluations with Nonrandomized Designs) statement for nonrandomized research.

## Abbreviations

The following abbreviations are used in this manuscript:

CAMS-R	Cognitive and Affective Mindfulness Scale—Revised
AEQ	Achievement Emotions Questionnaire
ERQ	Emotion Regulation Questionnaire
IRI	Interpersonal Reactivity Index
